# A Smart Water Bottle and Companion App (HidrateSpark 3) to Improve Bladder-Filling Compliance in Patients With Prostate Cancer Receiving Radiotherapy: Nonrandomized Trial of Feasibility and Acceptability

**DOI:** 10.2196/51061

**Published:** 2024-09-10

**Authors:** William Jin, Christopher Montoya, Benjamin James Rich, Crystal Seldon Taswell, Miguel Noy, Deukwoo Kwon, Benjamin Spieler, Brandon Mahal, Matthew Abramowitz, Raphael Yechieli, Alan Pollack, Alan Dal Pra

**Affiliations:** 1 Department of Radiation Oncology Jackson Memorial Hospital Miami, FL United States; 2 Department of Radiation Oncology Sylvester Comprehensive Cancer Center University of Miami Health Systems Miami, FL United States; 3 Department of Public Health Sciences Miller School of Medicine University of Miami Miami, FL United States; 4 Department of Internal Medicine McGovern Medical School University of Texas Health Science Center at Houston Houston, TX United States

**Keywords:** digital therapeutics, behavioral intervention, digital health, prostate cancer, radiation, smart water bottle, companion app, oncology, prostate, privacy, radiation therapy, bladder, compliance, smartphone-based behavioral intervention, mobile phone

## Abstract

**Background:**

Patients with prostate cancer undergoing radiation therapy (RT) need comfortably full bladders to reduce toxicities during treatment. Poor compliance is common with standard of care written or verbal instructions, leading to wasted patient value (PV) and clinic resources via poor throughput efficiency (TE).

**Objective:**

Herein, we assessed the feasibility and acceptability of a smartphone-based behavioral intervention (SBI) to improve bladder-filling compliance and methods for quantifying PV and TE.

**Methods:**

In total, 36 patients with prostate cancer were enrolled in a single-institution, closed-access, nonrandomized feasibility trial. The SBI consists of a fully automated smart water bottle and smartphone app. Both pieces alert the patient to empty his bladder and drink a personalized volume goal, based on simulation bladder volume, 1.25 hours before his scheduled RT. Patients were trained to adjust their volume goal and notification times to achieve comfortably full bladders. The primary end point was met if qualitative (QLC) and quantitative compliance (QNC) were >80%. For QLC, patients were asked if they prepared their bladders before daily RT. QNC was met if bladder volumes on daily cone-beam tomography were >75% of the simulation’s volume. The Service User Technology Acceptability Questionnaire (SUTAQ) was given in person pre- and post-SBI. Additional acceptability and engagement end points were met if >3 out of 5 across 4 domains on the SUTAQ and >80% (15/18) of patients used the device >50% of the time, respectively. Finally, the impact of SBI on PV and TE was measured by time spent in a clinic and on the linear accelerator (linac), respectively, and contrasted with matched controls.

**Results:**

QLC was 100% in 375 out of 398 (94.2%) total treatments, while QNC was 88.9% in 341 out of 398 (85.7%) total treatments. Of a total score of 5, patients scored 4.33 on privacy concerns, 4 on belief in benefits, 4.56 on satisfaction, and 4.24 on usability via SUTAQ. Further, 83% (15/18) of patients used the SBI on >50% of treatments. Patients in the intervention arm spent less time in a clinic (53.24, SEM 1.71 minutes) compared to the control (75.01, SEM 2.26 minutes) group (*P*<.001). Similarly, the intervention arm spent less time on the linac (10.67, SEM 0.40 minutes) compared to the control (14.19, SEM 0.32 minutes) group (*P*<.001).

**Conclusions:**

This digital intervention trial showed high rates of bladder-filling compliance and engagement. High patient value and TE were feasibly quantified by shortened clinic times and linac usage, respectively. Future studies are needed to evaluate clinical outcomes, patient experience, and cost-benefit.

**Trial Registration:**

ClinicalTrials.gov NCT04946214; https://www.clinicaltrials.gov/study/NCT04946214

## Introduction

### Background

Patients with prostate cancer (PCa) undergoing radiation therapy (RT) are asked to self-manage bladder volumes throughout their daily radiation treatments. A consistent and comfortably full bladder is important to (1) minimize treatment-related toxicity by decreasing radiation dose to adjacent normal organs, and (2) to potentially maximize treatment precision by decreasing prostate motion and improving target stabilization. However, dosimetric analyses have shown considerable intrapatient variation in bladder volumes during treatment [1-3]. This multifactorial issue limits the effectiveness of a radiation treatment plan by reducing the plan’s overall reproducibility.

Currently, there is no industry-standardized practice built into the management of an unfilled bladder. Some clinical practices have rigorous protocols in place, with standardized drinking and voiding intervals combined with pretreatment volume checks using bladder ultrasounds [4]. Yet bladder scan volumes vary by as much as 20% from daily cone-beam computerized tomography (CT) volumes, leaving much to be desired [5,6]. For practices that check bladder volumes with cone-beam CTs, patients with suboptimal bladder filling may need their treatment postponed until they fill their bladders appropriately, causing a preventable treatment delay. This translates to a loss of value for the patient and the clinician, and an identifiable inefficiency in the health care system.

### Prior Work

Prior attempts at maintaining consistent, comfortably filled bladders, as defined by the treatment planning CTs, have found little success. For example, in a previous clinical trial aimed at determining the best technique for maintaining consistent bladder volumes, a set of explicit instructions was given to patients where they were told to drink 300 ml of water 1 hour before radiation treatment or told to arrive with a full bladder [7]. They discovered that despite having bladder-filling protocols, about half the patients in both arms forgot to do *anything* and arrived with an empty bladder. Attempts at identifying a minimum volume required for consistent filling are highly variable [8], and shift away from the expectation of a personalized treatment experience. Noncompliance with bladder filling remains a common occurrence in the daily treatment of PCa [9,10]. Treating with empty bladders may increase the risk of toxicity, as a full bladder pushes away the parenchymal bladder dome and bowel superiorly, away from the high-dose radiation field. Additionally, the dose delivered to the rectum increased in patients with empty bladders [11], and other studies have found that at least 150 ml was needed in the bladder to meet dosimetric constraints for adjacent normal tissue [12-14].

Additionally, Grün et al [15] demonstrated that using biofeedback mechanisms for maintaining constant bladder volumes led to lower rates of significant (grade 2 or higher) acute genitourinary toxicities. In this current age, digital behavioral interventions found utility by improving health outcomes through promoting habitual change. The earliest successful trials that leveraged smartphone technology, or rather personal digital assistants, were directed at patients who were obese and at high risk for developing metabolic syndrome [16]. By assisting patients with the resources to effectively self-monitor their progress or regress and provide feedback, digital behavioral interventions empowered patients to take ownership of their health care. Subsequent studies found success even in socioeconomically disadvantaged populations, suggesting that the ubiquity of technology can disrupt socioeconomic health disparities [17]. The Pew Research Center’s 2019 survey revealed 81% of Americans own a smartphone, a significant increase from the 35% identified in 2011 [18]. Between the age 50 and 64 years brackets, this ~80% (4644/5733) smartphone ownership rate does not break down across gender, ethnicity, or income. However, smartphone ownership drops to 53% (1014/1914) in people aged older than 65 years. The primary demographic of men with PCa who receive RT is aged ≥50 years.

### Rationale for Study

Patients with PCa are generally a highly motivated population, compliant with dietary or lifestyle recommendations and actively engaged in their cancer care; yet the high rate of nonadherence to bladder-filling protocols leaves room for improvement. Sensory awareness of a bladder is usually limited to 2 states, full and not full. Patients with PCa may find it difficult to hold their bladder once they are aware it is full, which may be exacerbated by the high rate of comorbid prostatomegaly and lower urinary tract symptoms. Otherwise, patients normally do not have an awareness of a fractionally filled bladder (ie, 25% filled and 50% filled). Yet 50%-75% full is likely where the optimal filling of a bladder lies for radiation treatments. Therefore, we hypothesize that a smartphone-based behavioral intervention can motivate patients with PCa to optimally fill their bladders, reducing the need for reimaging while on the radiation treatment table and decreasing their overall time in a clinic.

## Methods

### Patient Selection

In total, 18 patients were prospectively enrolled in the intervention arm of a closed-access trial. They were eligible for enrollment if they were aged between 18 and 80 years, had American Joint Committee on Cancer 8th Edition Stage IA to IVA adenocarcinoma of the prostate requiring radiation treatment to the prostate, self-identified as “smartphone owners,” owned either an iPhone (iOS 13.0 or higher) or Android (version 5.0.1 or higher), and were English or Spanish speaking. Patients were excluded if they had any history of pre-existing chronic or acute urinary retention; had any history of kidney, urothelial tract, or bladder cancer; underwent prior pelvic radiation, prostatectomy, pelvic surgery, or penile augmentation; did not have a functional bladder; or did not have functional vision.

In addition, 18 patients who met eligibility criteria but declined to enroll in the trial were retrospectively selected as controls. Patients were age, stage, risk, and fractionation scheme matched to the interventional cohort. Additionally, only patients who received treatment within the same enrollment period as the interventional cohort were included in the control group. Outcomes data for the control group was only collected for quantifying patient and health system-centered value. Patients in this cohort received standard written or verbal instructions for bladder and bowel preparation.

### Ethical Considerations

Institutional review board approval was obtained (20200017) for this trial without any concerns. Informed consent was obtained from all patients at the first study visit. Patients were allowed to opt out at any time without penalty or fear of retaliation. All consumption volumes and times were synchronized to a cloud-based, remote patient monitoring platform based on anonymized research identification numbers (RIN). Generic user accounts were created for patients, with anonymized personal information (names were their RIN, emails were randomized emails generated by the institution). No institutional affiliations were displayed in the app or on the smart water bottle. Only patients’ RIN and smartphone make or model were collected in the cloud. No other personal health or self-identifying information was collected. While the remote patient monitoring platform was available for viewing to staff, they were instructed not to intervene if activity or usage decreased. Patients were not compensated for their participation in the trial, except for the smart water bottle intervention.

Patients were recruited at a single radiation oncology clinic at a National Cancer Institute–designated comprehensive cancer center. No selective patient sampling for study selection was performed. After being prescreened by our study coordinators, they were contacted either in person or via telephone to be introduced to this study. Informed consent was performed in person only at a subsequently determined study visit by our coordinators. No study advertisements or flyers were used.

### Intervention

**Figure 1:**
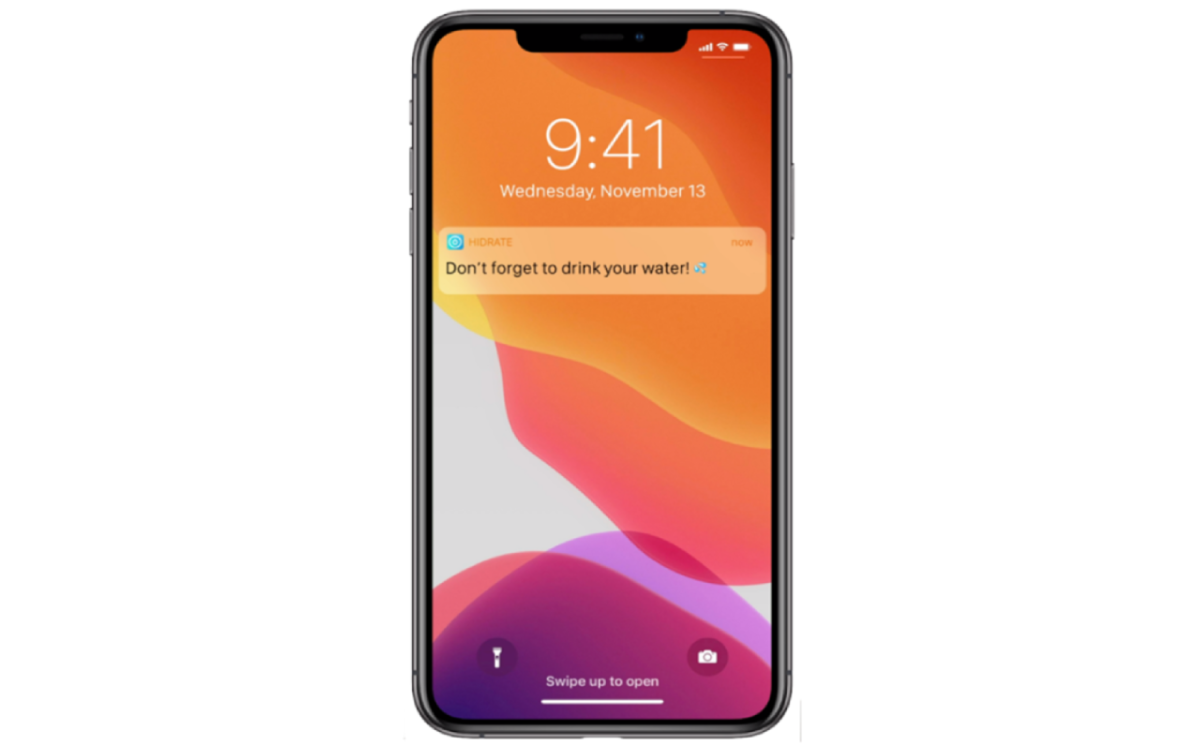
App will remind patients to drink water at preset times related to radiation treatment.

**Figure 2:**
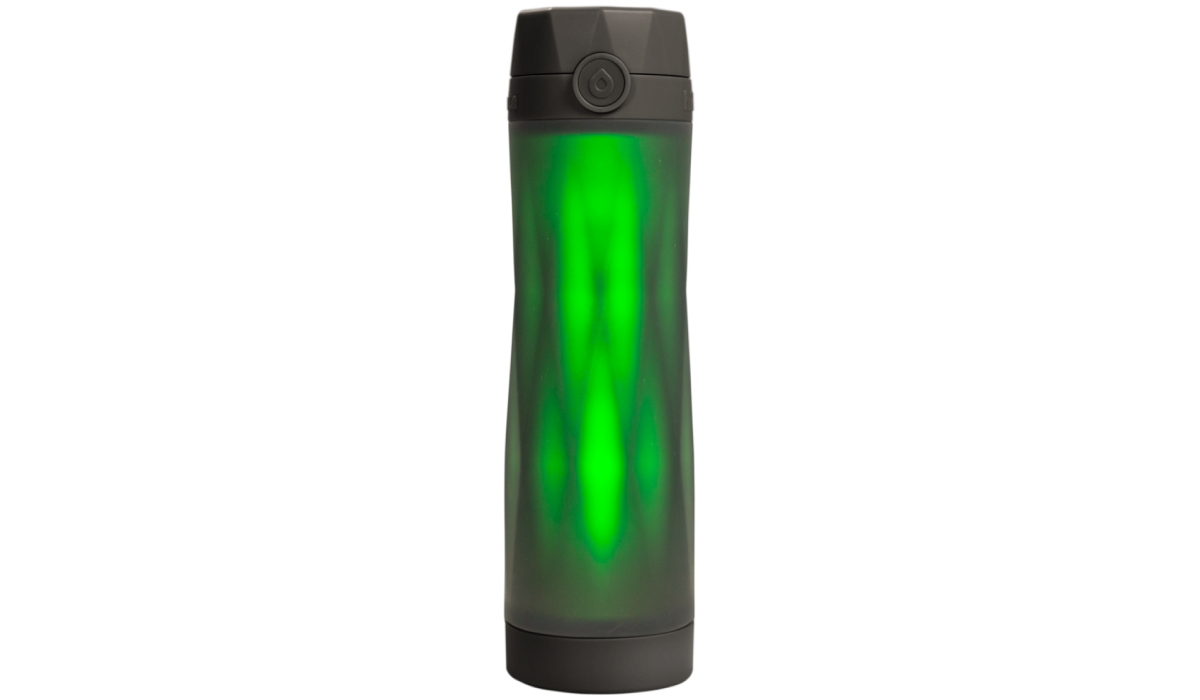
Smart water bottle will glow a bright fluorescent green color simultaneously with notifications.

The intervention is a combination of a smart water bottle, a black HidrateSpark 3, and its companion smartphone app (versions 2.4.1-3.0.3 used during the trial). Its volumetric quantification abilities were previously validated [19] and used in a large, multicenter, prospective trial to reduce the formation of kidney stones in patients with a history of recurrent nephrolithiasis [20]. The app synchronizes with the bottle when placed within Bluetooth range. Within the app, the timing of notifications and volume goals (VGs) for consumption can be programmed. At the appropriate time, the smartphone will send a notification reminding patients to void their bladders and begin drinking the VG (Figure 1). Simultaneously, the bottle glows a bright, fluorescent green to provide another visual reminder (Figure 2). Notifications will be sent every 15 minutes until the VG is met that day. Patients were encouraged to use the intervention daily.

### Study Scheme

**Figure 3:**
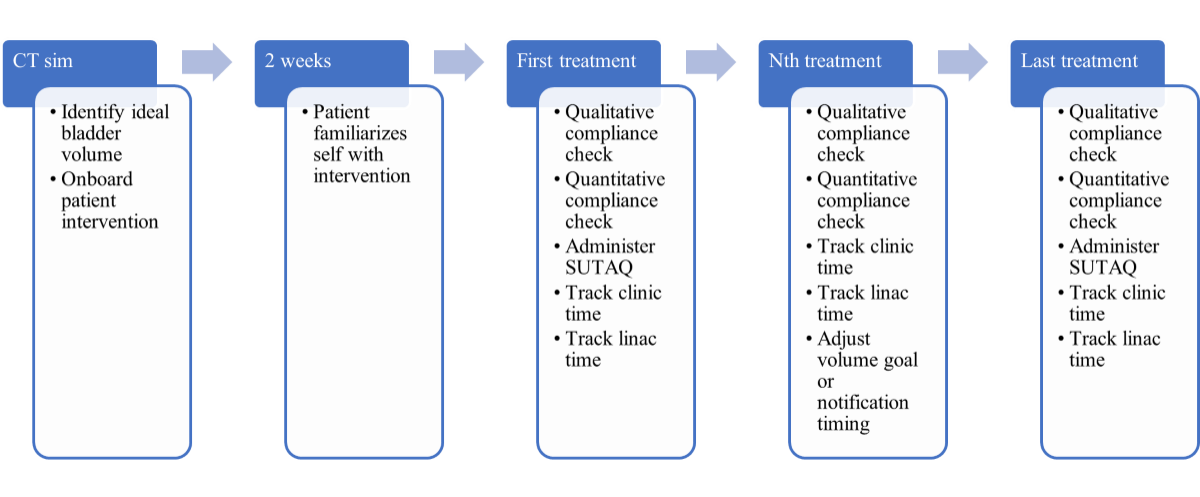
Study scheme. CT: computerized tomography; SUTAQ: Service User Technology Acceptability Questionnaire.

Patients were provided in-person information about this study between the initial clinic visit and CT simulation; they could be enrolled at any point up until CT simulation, the first step in the standard of care RT pathway. During this session, the patient’s anatomy is captured in a CT scan and then exported to a treatment planning system. A radiation oncologist then delineates targets for treatment and organs at risk for dose minimization. Informed consent was obtained in person from research coordinators. On the day of the CT simulation, patients were onboarded and trained to use the intervention (Figure 3). Smart water bottles were also given at no cost to patients. Trained staff reviewed the functions of the app, particularly on how to adjust notification timings and daily VGs. The initial VG was set to the volume of the bladder contour delineated on CT simulation. The initial notification times were set at 1 hour and 15 minutes before their treatment time.

On the first day of treatment, patients were administered an in-person questionnaire aimed at addressing the acceptability of digital technologies. During daily radiation treatments, patients were asked (in person) if they felt their bladders were adequately prepared. Staff were available during clinic hours to assist with any technical issues, such as adjustment of VGs, adjustment of notification times, bugs, malfunctions, and software issues. On the last day of treatment, patients were given the same in-person questionnaire.

### Primary End Point: Qualitative and Quantitative Bladder-Filling Compliance

Before every fraction of radiation, patients were asked if “[they] adequately prepared the bladder for treatment?” Qualitative compliance was measured by recording patients’ daily responses. Individual compliance status for this measure was met if ≥80% of responses were “yes.” Additionally, daily compliance was quantitatively assessed via 2 criteria. First, the patient must not be taken off the treatment table by the treating radiation oncologist after a review of initial CBCTs. Second, the bladder volume on the CBCT must be ≥75% of the bladder volume on the initial simulation CT. Individual compliance status for this measure was met if ≥80% (15/18) of patients met both criteria. The overall compliance rate for both measures was defined as the number of patients whose compliance status equals “yes” divided by the total number of patients in this study.

### Acceptability

Acceptability was evaluated using a modified version of the Service User Technology Acceptability Questionnaire [21]. This end point was met if mean scores in all domains of the Service User Technology Acceptability Questionnaire were ≥3 (SD 1.4142). Pre- and postintervention analyses were assessed via paired *t* tests (2-tailed). In addition, an in-person qualitative review of the patients’ responses was performed after the second questionnaire to improve acceptability in future trials and clinic integration.

### Engagement

**Figure 4:**
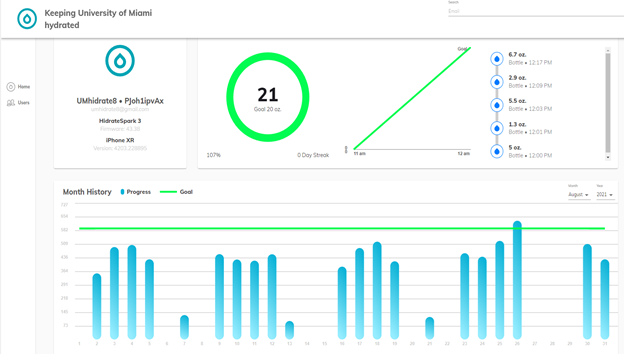
Remote patient monitoring platform showing example water bottle usage data. oz: ounce.

The engagement end point was met if >80% (15/18) of patients used their bottles on >50% of daily treatments. Engagement was tracked using the remote monitoring platform (Figure 4). Age at diagnosis, race, phone manufacturing year, median home price of patient’s zip code, distance from the cancer center, preferred language, and radiation fractionation scheme were evaluated for associations with poor engagement via binomial logistic regression.

### Quantifying Value—Patient Centered and Health System Centered

Patient value was quantified by the amount of time patients spent in the clinic, captured from the time that the patient signed into the check-in desk until the patient gets off the linear accelerator (linac). Health system value was quantified by the amount of time the patient spent on the linac, captured from when the patient was initially taken into the linac room to the time the patient gets off the linac table. To meet this end point, the prospective interventional cohort was contrasted with a retrospectively generated control group matched by age, stage, risk stratification, and radiation fractionation scheme. This end point was met if there was a statistically significant difference in mean times between the 2 groups.

### Statistical Analysis

All patients were prescreened from the radiation oncologic clinic for the prospective intervention arm and, if they met eligibility criteria, were invited to participate. To calculate the appropriate sample size, we used a historical bladder-filling compliance rate of 50%, based on data presented by Braide et al [7]. We anticipate the intervention will provide a 30% improvement over historical controls, thus requiring a sample size of 16 men. However, we aimed to recruit 18 men, which would allow for a 10% dropout or noncompliance rate, this would leave at least 16 evaluable patients, which would achieve 80% statistical power to detect a difference of 30% using a 1-sided binomial exact test with a 5% significance level.

Our primary end point, consisting of both qualitative and quantitative components, was assessed via descriptive statistics. To meet the end point, both components required ≥80% compliance. For acceptability, pre- and postintervention analyses were assessed via paired *t* tests. The engagement was similarly evaluated via descriptive statistics, where the end point was met if >80% (15/18) of patients used their bottles on >50% of daily treatments. Secondary analyses of engagement were evaluated by converting engagement (>50% daily use) into a binary categorical variable. This was set as the dependent variable, and age at diagnosis, race, phone manufacturing year, median home price of patient’s zip code, distance from the cancer center, preferred language, and radiation fractionation scheme were set as independent variables. Subsequently, univariate logistic regression was performed and statistically significant independent variables would be included in a multivariate model. Patient-centered and system-centered values were evaluated by 2-sample *t* tests, using time spent as the continuous variable.

For all statistics, *P*<.05 was considered significant. Data were analyzed using IBM SPSS (version 23.0.0.2; IBM Corp).

## Results

### Patient Demographics

Between June 6, 2021, and June 15, 2022, 18 men were enrolled in a single-arm, phase zero pilot study to evaluate a digital therapeutic for improving bladder-filling compliance during PCa radiotherapy. Most patients were English-speaking Hispanic-White men with unfavorable intermediate to high-risk PCa (Table 1). The most common fractionation scheme received was split evenly between 36.25 Gy in 5 fractions and 80 Gy in 40 fractions. The interventional cohort did not significantly differ from the retrospective control group in terms of race or ethnicity, preferred language, stage, risk stratification, or fractionation scheme.

**Table 1:** Patient demographics.

		Intervention	Control	*P* value	
Age at diagnosis (years), mean (SD)		64.94 (9.67)	68.00 (8.12)	.31	
**Race or ethnicity, n (%)**					.34
	Black	3 (16.7)	1 (5.6)		
	Hispanic White	10 (55.6)	14 (77.8)		
	Non-Hispanic White	5 (27.8)	3 (16.7)		
**Preferred language, n (%)**					.08
	English	14 (77.8)	9 (50)		
	Spanish	4 (22.2)	9 (50)		
**American Joint Committee on Cancer 8th Edition Staging, n (%)**					.21
	II-A	2 (11.1)	2 (11.1)		
	II-B	6 (33.3)	2 (11.1)		
	II-C	4 (22.2)	7 (38.9)		
	III-A	4 (22.2)	2 (11.1)		
	III-C	1 (5.6)	5 (27.89)		
	IV-A	1 (5.6)	0 (0)		
**Risk stratification, n (%)**					.84
	Low	2 (11.1)	1 (5.6)		
	Favorable intermediate	4 (22.2)	3 (16.7)		
	Unfavorable intermediate	5 (27.8)	7 (38.9)		
	High	7 (38.9)	7 (38.9)		
**Radiation fractionation scheme, n (%)**					≥.99
	36.25 Gy in 5 fractions	7 (38.9)	7 (38.9)		
	70.2 Gy in 26 fractions	4 (22.2)	4 (22.2)		
	80 Gy in 40 fractions	7 (38.9)	7 (38.9)		

### Primary End Point: Feasibility in Assessing Qualitative and Quantitative Bladder-Filling Compliance

Both qualitative and quantitative end points for bladder-filling compliance were met. Qualitatively, 18 out of 18 (100%) patients stated they prepared their bladders on 375 out of 398 (94.2%) daily radiation treatments. In addition, 16 out of 18 (89%) patients attained quantitative compliance on aggregate 341 out of 398 (85.7%) fractions.

### Acceptability

Overall, patients were accepting of the intervention (Table 2). There were minimal concerns for privacy issues (mean score 4.33, SD 0.97). Patients believed there were perceived benefits from the intervention (mean score 4.00, SD 0.918), were satisfied with the intervention (mean score 4.56, SD 0.56), and noted high usability (mean score 4.24, SD 0.62). In addition, there was a statistically significant association between feeling less concerned about their health between pre- and postintervention scores (*P*=.02).

**Table 2:** Service user technology acceptability questionnaire results.

Domain and question text			Preintervention score, mean (SD)		Postintervention score, mean (SD)		*P* value
**Privacy**							
	The kit I received has not invaded my privacy.	4.056 (0.99)		4.556 (0.62)		.06	
	I am not concerned about the level of expertise of the individuals who monitor my health status via the kit.	4.000 (1.14)		4.056 (1.39)		.86	
	The kit does not make me worried about the confidentiality of the private information being exchanged through it.	4.333 (0.69)		4.389 (0.70)		.79	
**Perceived benefits**							
	This kit has made it easier to get in touch with my health care professionals.	3.667 (0.97)		4.056 (0.80)		.25	
	The kit I received has increased my access to care.	4.000 (0.77)		4.111 (0.67)		.54	
	The kit I received has helped me improve my health.	3.778 (0.81)		4.056 (0.73)		.33	
	This kit has helped me to improve my health.	4.056 (0.80)		4.278 (0.67)		.33	
	I do not feel anxious or nervous about the required bladder and rectal preparation for radiation treatment.	3.667 (1.28)		3.833 (1.15)		.64	
	The kit has allowed me to be less concerned about my health and social care.	3.111 (1.08)		3.889 (1.08)		.02	
	The kit has made me more actively involved in my health.	4.167 (0.92)		4.222 (0.73)		.83	
	This kit can certainly be a good addition to my regular health or social care.	4.444 (0.51)		4.389 (0.70)		.77	
	This kit has allowed me to be less concerned about my health status.	2.611 (1.46)		2.944 (1.11)		.38	
	The kit allows the people looking after me, to better monitor me and my condition.	4.500 (0.62)		4.278 (0.70)		.16	
**Satisfaction**							
	I am satisfied with the kit I received.	4.389 (0.61)		4.556 (0.51)		.33	
	This kit can be and should be recommended to people in a similar condition to mine.	4.500 (0.62)		4.556 (0.62)		.67	
**Usability**							
	The kit I received has been explained to me sufficiently.	4.500 (0.62)		4.611 (0.61)		.43	
	The kit can be trusted to work appropriately.	4.278 (0.83)		4.333 (0.84)		.77	
	The kit has not made me feel uncomfortable, either physically or emotionally.	4.278 (1.13)		4.667 (0.49)		.17	
	This kit interferes with the continuity of care I receive.	2.000 (1.08)		2.000 (1.19)		.99	
	The kit I received has not interfered with my everyday routine.	4.167 (1.29)		4.611 (0.50)		.18	

### Engagement

The minimum engagement end point was met, as 15 out of 18 (83%) patients used the intervention on >50% of treatments throughout the trial. Additionally, 9 out of 18 (50%) patients used the bottle on 100% of treatments while 12 out of 18 (67%) patients used it on >85% of treatments. None of the a priori variables were significantly associated with poor engagement in univariate analysis, so a multivariate model was not generated. Specifically, the independent variables were age at diagnosis (*P*=.18), self-identified race (*P*=.82), median home price of zip code (*P*=.13), distance from cancer center (*P*=.10), preferred language (*P*=.87), and radiation fractionation (*P*=.34). Of the 3 patients who did not meet the minimum engagement criteria, 2 stated they needed a reminder to keep the physical water bottle nearby, and one encountered too many technical issues with water bottle refilling.

### Feasibility in Quantifying Value—Patient Centered and Health System Centered

**Figure 5:**
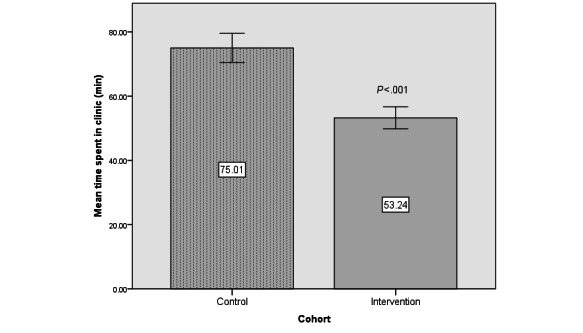
Mean time spent in minutes (SEM) in clinic between intervention and control cohorts.

**Figure 6:**
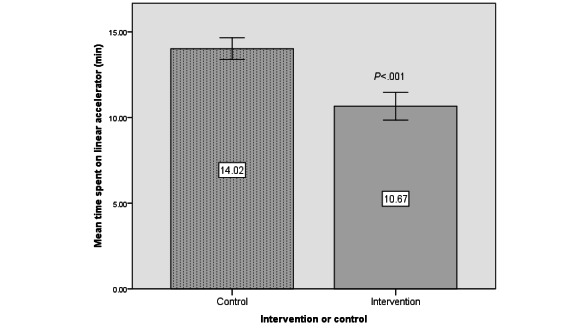
Mean time spent in minutes (SEM) on the linear accelerator between intervention and control cohorts.

Patients in the intervention arm spent less time in the clinic (53.24, SEM 1.71 minutes) compared to the control (75.01, SEM 2.26 minutes) group (*P*<.001, Figure 5). Similarly, the intervention arm spent less time on the linac (10.67, SEM 0.41 minutes) compared to the control (14.19, SEM 0.32 minutes) group (*P*<.001, Figure 6).

When looking at the data more granularly, patients with empty bladders (n=43) spent significantly more time (75.14 vs 50.59 minutes, *P*=.007) in the clinic than patients who came with full bladders (n=355, Table S1 in Multimedia Appendix 1). Similarly, these same patients spent nearly twice as long on the linac (21.63 vs 12.50 minutes, *P*<.001, Figure S1 in Multimedia Appendix 1). However, the presence of stool in the rectum had more impact on clinic time. Expectedly, the presence of both an unprepared bladder and rectum led to the longest time spent in the clinic at 93.13 minutes (Figure S2 in Multimedia Appendix 1).

## Discussion

### Principal Results

This prospective study aimed to assess the feasibility and acceptability of a smart water bottle and companion app as an intervention to improve bladder-filling compliance in patients with PCa receiving RT. We showed that the intervention can be feasibly integrated into the clinic, retains high engagement, and was perceived by patients with high acceptability. In addition, the intervention was effective at reducing wasted value for the patient and the clinic, compared to matched controls.

### Results in Context of Prior Work

To our knowledge, this is the only prospective study that evaluated a digital intervention for bladder filling in patients with PCa undergoing radiotherapy. However, multiple institutions have identified the benefits of delivering scalable care using mobile apps and started preliminary, pilot, and feasibility studies. For example, the Karolinska Institute reported on their study of the Interaktor app for their patients with PCa undergoing RT [22]. This app collected and triaged patient symptoms during RT, sent alerts to managing health care providers, and provided self-care advice. It was well received by patients (n=75), and daily symptom reporting was high, with 83%-87% adherence or engagement reported [22,23]. In addition, Thomas Jefferson University evaluated the feasibility and acceptability of its Strength Through Insight app, a tool that assessed electronic patient-reported outcomes during RT using a validated symptom questionnaire [24]. Similarly, we found that our patients were not only accepting and enthusiastic about using the intervention, but many believed that smartphone integration into their clinical care was long overdue. Digital consumer experiences in other industries may be shifting expectations for similar services in health care [25-27].

However, many barriers remain that prevent seamless digital therapeutic integration in the clinic. Key stakeholder buy-in is missing. Identifying the concerns of all stakeholders is necessary to create and mimic the infrastructure supporting the pharmaceutical industry. Our trial quantified the value of poor prostate radiotherapy preparation for both the clinic *and* the patient. Whereas prior studies only sought to identify waste that impacts health care spending, we also quantified patient value to prevent 0-sum game situations. For example, 4 separate institutions used bladder scanners to increase the probability of an adequately filled bladder on daily cone-beam CT [4,7,28,29], thereby potentially reducing the time wasted on the linac for checking unprepared bladders. However, it is often at the cost of the patient’s experience. If a patient arrives for his PCa treatment with an unprepared bladder, he still needs to spend time at the clinic fixing the issue, regardless of how much time was saved in the treatment room. Often, patients may feel that they failed their responsibility for adequate preparation, creating a sense of anxiety and a devalued overall experience [30].

Our study suggests that network effects may have a large role in engagement, a critical component of a successful digital intervention [31]. The goal is to smoothly embed itself into the daily lives of patients, continually analyze recorded data, and interject behavioral interventions when needed. Failures arise when subclinical usage occurs [32]. This study did not isolate patient experiences; those with the intervention were waiting for their treatments in the same waiting room as nontrial patients. Anecdotally, 2 of the patients with nearly 100% engagement felt empowered that they could use the intervention to improve their bladder-filling compliance, especially when they saw another patient struggling. The 2 patients who did not meet the engagement end point also had interesting similarities. Both were actively working and delegated the task of keeping the water bottle nearby to their spouses.

Finally, while the purpose of this trial was to evaluate an intervention on bladder-filling compliance, our data suggests there were nearly twice as many patients with poor rectal preparation than poor bladder preparation (Figures S1 and S2 in Multimedia Appendix 1). This suggests that to maximize time-based value metrics, better strategies for rectal preparation are also required.

### Limitations

This study was limited by its design as a pilot study, specifically in extrapolating conclusions for the value-based end points using retrospectively matched controls. Specifically, quantitative and qualitative compliance data were only collected in the intervention arm. Additionally, interpreting the end points regarding acceptability may be confounded by patients who self-select as participants in a digital interventional trial. Designing apps agnostic to digital literacy is critical for ubiquitous adoption. The patient population may be limited in its diversity, as patients were enrolled at a private, National Cancer Institute-designated cancer hospital in South Florida. Quantification of digital literacy was not performed, as the intent of this study was to assess the technical, multi-stakeholder value, and subjective acceptance of the intervention, and this study would otherwise be underpowered.

### Conclusions

A smart water bottle and companion app can be feasibly integrated into a radiation oncology clinic for patients with PCa. Patients are accepting of this digital intervention, with minimal concerns for privacy issues. It is crucial to quantify value for all stakeholders (patients, clinical team, economics) and identify 0-sum situations. This digital intervention has the potential to enhance value for all stakeholders concerned.

## Appendix

Multimedia Appendix 1 Quantifying value for the patient and for the health system, time spent on linear accelerator, and time spent in clinic.

## Abbreviations

CT: computerized tomography
linac: linear accelerator
PCa: prostate cancer
PV: patient value
QLC: qualitative compliance
QNC: quantitative compliance
RIN: research identification number
RT: radiation therapy
SBI: smartphone-based behavioral intervention
SUTAQ: Service User Technology Acceptability Questionnaire
TE: throughput efficiency
VG: volume goal

## Data Availability

The data sets generated or analyzed during this study are not publicly available due to secondary analyses currently being run on them for another paper but are available from the corresponding author upon reasonable request.
