# Supplementary material for: A Smart Water Bottle and Companion App (HidrateSpark 3) to Improve Bladder-Filling Compliance in Patients With Prostate Cancer Receiving Radiotherapy: Nonrandomized Trial of Feasibility and Acceptability
Source: JMIR Cancer. 2024 Sep 10;10:e51061. doi: 10.2196/51061 (PMC11422727; doi:10.2196/51061)
Supplement: Multimedia Appendix 1 [file cancer_v10i1e51061_app1.docx]

**Table S1.** Quantifying value for the patient and for the health system.

|  | | Minutes Spent in Clinic | |  | Minutes Spent on Linear Accelerator | |  |
| --- | --- | --- | --- | --- | --- | --- | --- |
|  |  | Mean | n | P-value | Mean | n | P-value |
| Not Prepared: Rectum & Bladder | Yes | 93.13 | 23 | 0.004 | 25.01 | 23 | 0.003 |
|  | No | 50.79 | 375 |  | 12.78 | 375 |  |
| Not Prepared: Rectum Only | Yes | 72.97 | 97 | <0.001 | 21.30 | 97 | <0.001 |
|  | No | 46.88 | 301 |  | 10.97 | 301 |  |
| Rectum: Gas | Yes | 66.83 | 72 | <0.001 | 20.87 | 72 | 0.008 |
|  | No | 50.24 | 326 |  | 11.86 | 326 |  |
| Rectum: Stool | Yes | 78.48 | 44 | 0.209 | 21.12 | 44 | 0.007 |
|  | No | 50.10 | 354 |  | 12.54 | 354 |  |
| Not Prepared: Bladder Only | Yes | 74.61 | 57 | 0.006 | 20.88 | 57 | <0.001 |
|  | No | 49.67 | 341 |  | 12.25 | 341 |  |
| Bladder: Too Empty | Yes | 75.14 | 43 | 0.007 | 21.63 | 43 | <0.001 |
|  | No | 50.59 | 355 |  | 12.50 | 355 |  |
| Bladder: Too Full | Yes | 71.81 | 16 | 0.969 | 16.85 | 16 | 0.016 |
|  | No | 52.46 | 382 |  | 13.35 | 382 |  |

**Figure S1.** Time spent on linear accelerator.

**Figure S2.** Time spent in clinic.
